# Integrated analysis reveals potential long non-coding RNA biomarkers and their potential biological functions for disease free survival in gastric cancer patients

**DOI:** 10.1186/s12935-019-0846-6

**Published:** 2019-05-07

**Authors:** Canchang Cheng, Qicai Wang, Minggu Zhu, Kelong Liu, Zhiqiao Zhang

**Affiliations:** 1 Department of Internal Medicine, The Affiliated Chencun Hospital of Shunde Hospital, Southern Medical University, Shunde District, Guangdong China; 2 Department of General Surgery, The Affiliated Chencun Hospital of Shunde Hospital, Southern Medical University, Shunde District, Guangdong China

**Keywords:** Long non-coding RNA, lncRNA, Gastric cancer, Disease free survival, Prognostic signature

## Abstract

**Background:**

Increasing evidences supported the association between long non-coding RNA (lncRNA) and disease free survival in gastric cancer (GC) patients. The purpose of the current study was to construct and verify a noninvasive preoperative predictive tool for disease free survival in GC patients.

**Methods:**

There were 265 and 300 GC patients in model dataset and validation dataset respectively. The associations between the lncRNA biomarkers and disease free survival were evaluated by univariate and multivariate Cox regression.

**Results:**

Thirteen lncRNA biomarkers (GAS5-AS1, AL109615.3, KDM7A-DT, AP000866.2, KCNJ2-AS1, LINC00656, LINC01777, AC046185.3, TTTY14, LINC01526, LINC02523, LINC00592, and C5orf66) were identified as prognostic biomarkers with disease free survival. These thirteen lncRNA biomarkers were combined to construct a prognostic signature for disease free survival. The C-indexes of the current predictive signature in model cohort were 0.849 (95% CI 0.803–0.895), 0.859 (95% CI 0.813–0.905) and 0.888 (95% CI 0.842–0.934) for 1-year, 3-year and 5-year disease free survival respectively. Based on thirteen-lncRNA prognostic signature, patients in model cohort could be stratified into high risk group and low risk group with significant different disease free survival rate (hazard ratio [HR] = 7.355, 95% confidence interval [CI] 4.378–12.356). Good reproducibility of thirteen-lncRNA prognostic signature was confirmed in an external validation cohort (GSE62254) with HR 3.919 and 95% CI 2.817–5.453. Further analysis demonstrated that the prognostic significance of thirteen-lncRNA prognostic signature was independent of other clinical characteristics.

**Conclusions:**

In conclusion, a simple noninvasive prognostic signature was established for preoperative prediction of disease free survival in GC patients. This prognostic signature might predict the individual mortality risk of disease free survival without pathological information and facilitate individual treatment decision-making.

**Electronic supplementary material:**

The online version of this article (10.1186/s12935-019-0846-6) contains supplementary material, which is available to authorized users.

## Introduction

As a serious challenge to public health, gastric cancer (GC) is the fifth most common cancer and the third leading cause for cancer associated mortality in the world [1]. It was estimated that there were approximately 1,033,701 GC patients occurred and 782,685 GC patients died in 2018 [1]. Despite the improvements of diagnosis and treatments, the prognosis of GC patients remains undesirable [2, 3]. The TNM stage system of the American Joint Committee on Cancer (AJCC) was insufficient for prognostic prediction of GC patients [4, 5]. Increasing evidences demonstrated that the molecular biomarkers were helpful for improvement of prognostic prediction and early identification of GC patients with high mortality risk [6–8]. Thus, it is necessary to develop a valuable preoperative prognostic signature to identify GC patients with high mortality risk and improve the clinical treatment decision.

Long non-coding RNAs (lncRNAs) are RNAs that length range from 200 nucleotides to multiple kilobases but lack of protein-coding function [9]. Emerging evidences have revealed that lncRNAs could provide valuable prognostic information for GC patients [10–12]. Several studies have developed lncRNA-based prognostic signatures for disease free survival (DFS) in GC patients [13–15]. However, these lncRNA-based prognostic signatures had several limitations for preoperative prediction of disease free survival: Firstly, the calculation formulas of these lncRNA-based prognostic signatures were too complex for clinical application. Secondly, the prognostic significances of absolute scores of these previous prognostic signatures were difficult to understand and interpret for users without medical knowledge. Thirdly, these three prognostic signatures lacked external validation. Fourthly, Tian et al. constructed a lncRNA-based prognostic signature for 3-year DFS by combination of data of gene expression and pathological parameters. However, for patients with advanced GC cancer or who were unwilling to undergo surgery, the pathological parameters were unobtainable for calculation of prognostic signature and thus seriously limited the clinical application of this prognostic signature. Thus, it is necessary to develop and validate a simple noninvasive signature for preoperative prediction of prognosis in GC patients.

Nomogram was a method of displaying calculation results by percentage scale diagram and was used for prognostic prediction in different cancer patients [8, 16]. Nomogram could easily attain the predictive percentage of study outcome without complex calculation. Therefore, to construct a simple prognostic signature for disease free survival in GC patients, the current study developed and validated a prognostic signature by using nomogram method. We carried out the current study and reported the results in accordance with the guidelines of Transparent Reporting of a multivariable prediction model for individual prognosis or diagnosis (TRIPOD) [17].

## Materials and methods

### Protocol approval

The study datasets in the current study were obtained from The Cancer Genome Atlas (TCGA) database (https://cancergenome.nih.gov/) and the Gene Expression Omnibus (GEO) database (https://www.ncbi.nlm.nih.gov/gds/). The current study downloaded and analyzed the study datasets according to the data policies of TCGA database and GEO database. The study datasets obtained from TCGA database and GEO database were fully anonymous and therefore the ethics approval was not required.

### The model dataset

**Fig. 1:**
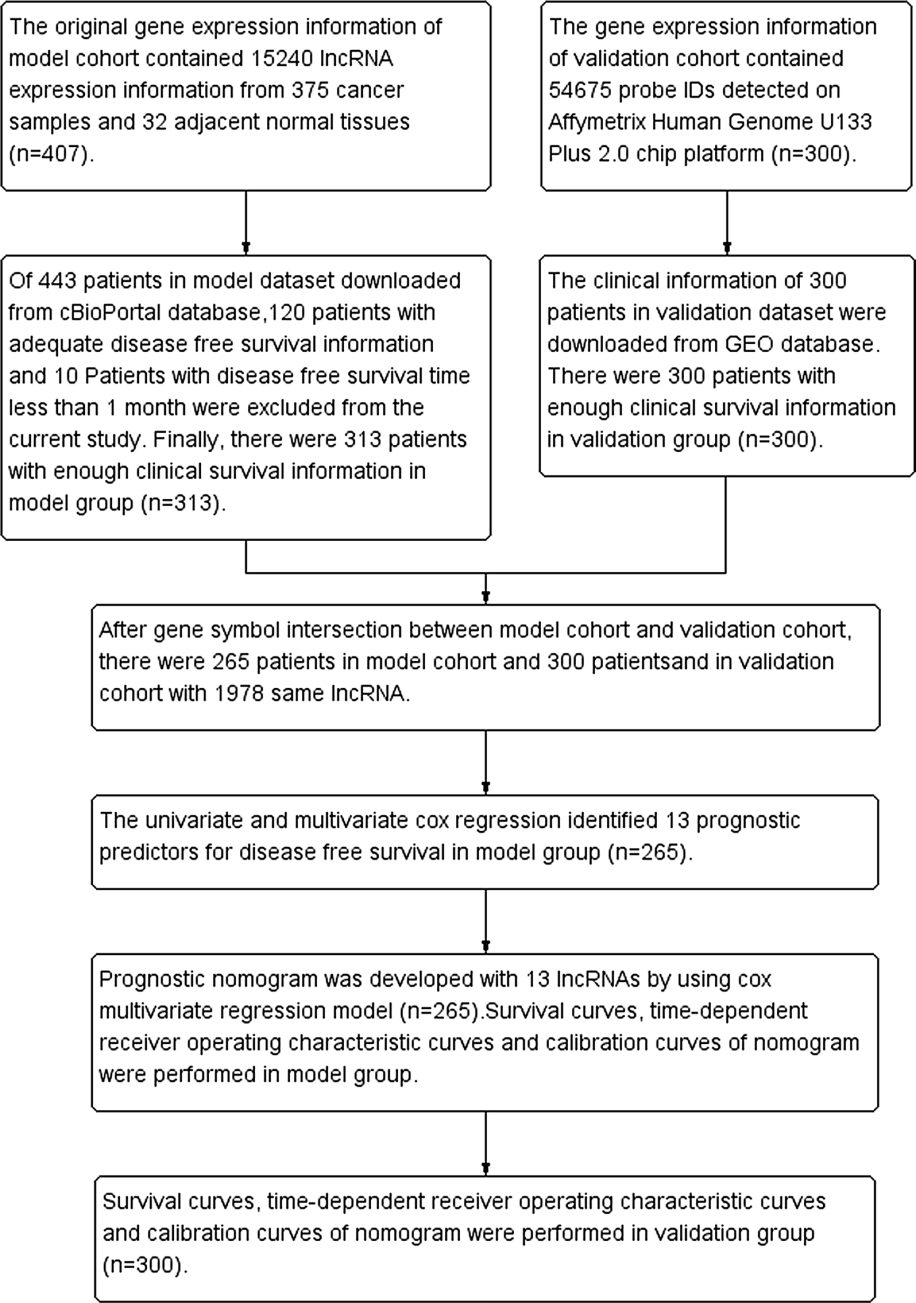
The flowchart in the current study. TCGA, The Cancer Genome Atlas

The model dataset was downloaded from TCGA database (https://tcga-data.nci.nih.gov/docs/publications/tcga/). The gene expression values were generated by using the Illumina HiSeq 2000 RNA Sequencing platform. In the current study, the selected lncRNA IDs were defined according to GENCODE Version 29 (https://www.gencodegenes.org/human/). Finally, there were 14,449 lncRNAs obtained from 375 tumor specimens and 32 normal specimens in model dataset. The original gene expression counts were converted to 0 for low expression and 1 for high expression according to the median values of original gene expression values. The clinical information of 443 GC patients in model dataset were downloaded from cBioPortal database (http://www.cbioportal.org/datasets). The GC patients without disease free survival information were excluded from the current study (n = 120). In order to avoid or reduce the interferences of confounding factors, there were 10 GC patients excluded from the current study because disease free survival time less than 1 month (n = 10). After interaction between gene dataset and clinical dataset, there were 265 GC patients with gene expression information and survival information included in the model cohort (Fig. 1). The maximum DFS time was 122.21 months and the minimum DFS time was 1.02 month in model cohort. The study time was from January 13, 2002 to November 24, 2014. The missing data were coded as “NA” in the model dataset. The mean ± standard deviation (SD) age of GC patients in the model cohort was 64.4 ± 10.6 years. Ninety-eight (37.0%) patients out of 265 GC patients died within the follow-up period (mean ± SD: 618 ± 564 days).

### The validation dataset

We explored and identified potential study datasets in GEO database according to the following criteria: (1) gene expression values detected by using the Affymetrix Human Genome U133 Plus 2.0 array; (2) DFS time and DFS status were provided in the corresponding studies; (3) patient number more than 100. Finally, we identified GSE62254 as an independent external validation dataset [18]. The gene expression information and corresponding clinical information were downloaded from the GEO database (http://www.ncbi.nlm.nih.gov/geo/). There were 300 GC patients with gene expression information and clinical information from GSE62254 (https://www.ncbi.nlm.nih.gov/geo/query/acc.cgi?acc=GSE62254). The gene expression information was detected on Affymetrix Human Genome U133 Plus 2.0 chip platform. The patient selection flow chart was described in Fig. 1. The Affymetrix HG-U133 Plus 2.0 probe set IDs were mapped to the Ensembl gene IDs by using the platform background file of GPL570 (https://www.ncbi.nlm.nih.gov/geo/query/acc.cgi?acc=GPL570).

Based on the lncRNA IDs defined in GENCODE Version 29 (https://www.gencodegenes.org/human/), 1978 lncRNAs with corresponding gene symbols from 300 GC patients were extracted and analyzed for further survival study.

### Development and validation of the prognostic nomogram

The prognostic nomogram and the corresponding calibration plots were generated by using “rms” package of R software. Calibration plots were performed to evaluate the predictive performance of the prognostic nomogram. The predicted survival and observed survival were plotted on the x-axis and y-axis respectively. The 45-degree line represented the best predictive curve. Time-dependent ROC curves were conducted to assess the predictive performance of the prognostic nomogram by using the “pROC” package.

### The decision curve analysis

The decision curve analysis (DCA) was performed to evaluate the clinical utility of the prognostic nomogram for disease free survival. The DCA is a method for evaluation and comparison of the predictive value between different prediction models [19–21]. The x-axis of DCA represented the percentage of threshold probability, and the y-axis represented the net benefit of the predictive model. The net benefit was calculated according to the following formula: Net benefit = (True positives/n) − (False positives/n) * (p_t_/(1 − p_t_).

### Functional enrichment analyses

To explore the potential biological functions of lncRNAs included in the prognosis signature, the co-expressed mRNA were obtained through the model dataset according to the thresholds of *P* value < 0.05 and |spearman correlation coefficient| > 0.5. Functional enrichment analyses of these co-expressed mRNAs were performed by using the Database for Annotation, Visualization and Integrated Discovery (DAVID, https://david.ncifcrf.gov/) [22].

### Statistical analysis

Continuous variables were displayed as mean ± standard deviation (SD). Continuous variables were compared by *t* test or Mann–Whitney U test as appropriate. Categorical variables were compared by using Chi squared test or Fisher’s exact test as appropriate. To explore the potential associations between lncRNAs and DFS, univariate Cox regression analyses and multivariate Cox regression analyses were performed to identify the prognostic biomarkers for development of prognosis signature. The GC patients were divided into two subgroups according to the scores generated by the prognosis signature. Kaplan–Meier analysis was used to compare the difference of DFS between high risk group and low risk group. The predictive performance and clinical utility of prognostic signatures were evaluated by using the Harrell’s concordance index and time-dependent receiver operating characteristic (ROC) curve. The statistical analyses in the present study were conducted by SPSS Statistics 19.0 (SPSS Inc., an IBM Company) and R software (version 3.4.5). The R packages, including “pROC”, “plyr”, “rms”, “survival”, “timeROC “ and “glmnet “, were used as needed in the current study. *P* value < 0.05 was defined as statistically significance in the current study.

## Results

### Study cohort

The flow chart of patient selection in the current study was showed in Fig. 1. There were 265 GC patients in the model group (Additional file 1) and 300 GC patients in the validation group (Additional file 2). There were 98 (37.0%) patients out of 265 patients died within the follow-up period in the model group, whereas there were 161 (53.7%) patients out of 300 patients died within the follow-up period in the validation group. The basic clinical characteristics of GC patients in the model group and validation group were presented in Table 1.

**Table 1:** The clinical features of gastric cancer patients in the model group and validation group

	Model group (n = 265)	Validation group (n = 300)	*P* value
Death [n (%)]	98 (37.0)	161 (53.7)	< 0.001
Survival time (mean ± SD, month)	20.6 ± 18.8	33.7 ± 29.8	0.008
Age (mean ± SD, year)	64.4 ± 10.6	61.9 ± 11.4	< 0.001
Male [(n) %]	175 (66.0)	199 (66.3)	0.941
AJCC Stage (IV/III/II/I/NA)	23/103/91/41/7	77/95/98/30/0	< 0.001
AJCC PT (T4/T3/T2/T1/NA)	66/121/62/16/0	21/91/188/0/0	< 0.001
AJCC PN (N4/N3/N2/N1/N0/NA)	4/49/55/67/88/2	0/51/80/131/38/0	0.011
AJCC PM (M2/M1/M0/NA)	9/13/243/0	0/27/273/0	0.869
Targeted molecular therapy (yes/no/NA)	78/79/108	NA	
Radiation treatment adjuvant (yes/no/NA)	0/155/110	NA	
History other malignancy (yes/no/NA)	6/259/0	NA	
Barretts esophagus (yes/no/NA)	12/151/102	NA	
H pylori infection (yes/no/NA)	15/117/133	NA	

Continuous variables were compared by t-test or Mann–Whitney U test as appropriate; categorical variables were compared by Chi squared test or Fisher’s exact test as appropriate

NA, missing data; SD: standard deviation; AJCC: American Joint Committee on Cancer

### Development of prognostic signature

**Fig. 2:**
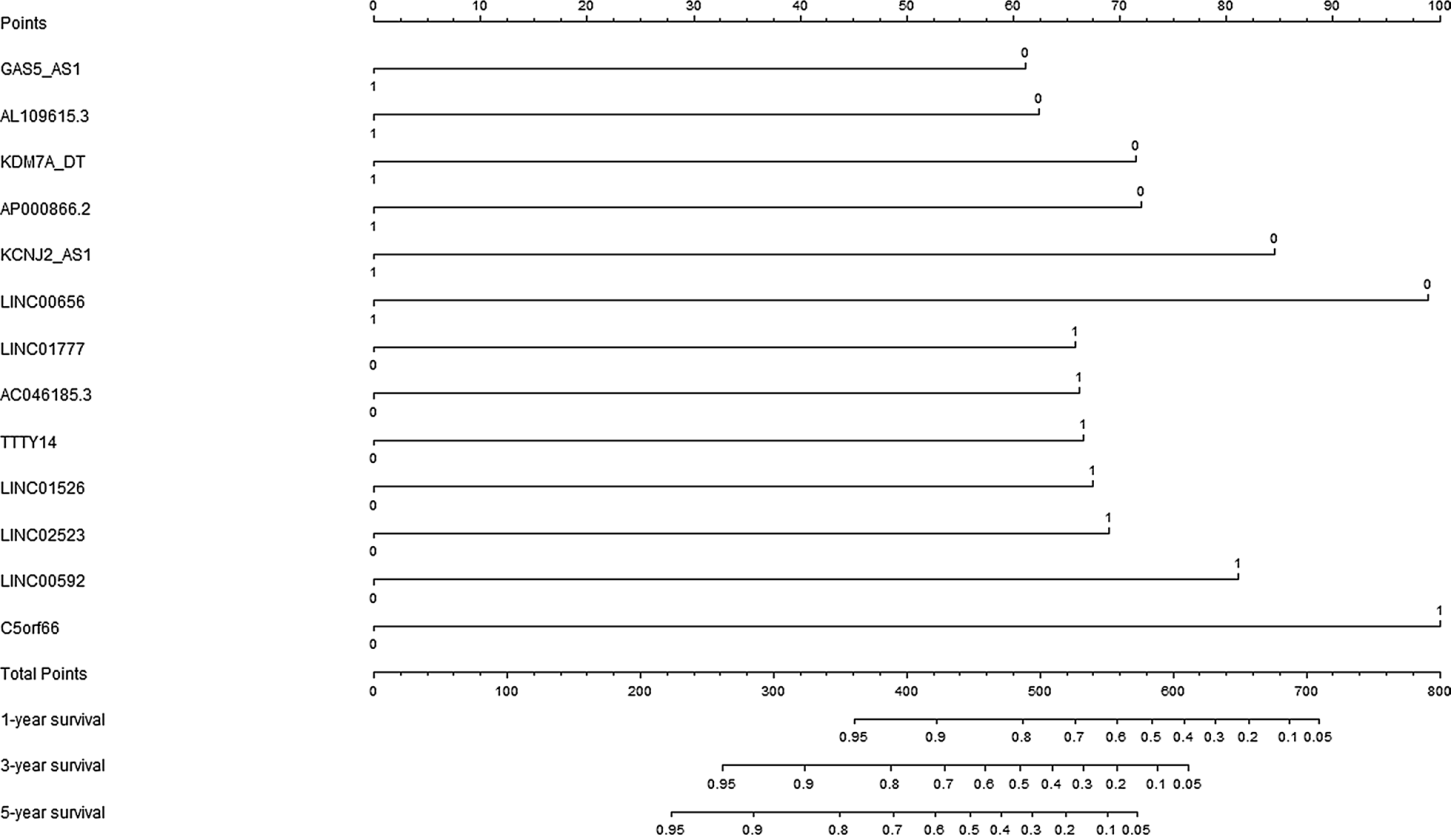
The prognostic signature for prediction of disease free survival in gastric cancer patients

The univariate Cox proportional regression analyses were carried out to explore the potential lncRNA predictors for disease free survival in GC patients. There were 249 lncRNAs that were significantly related with DFS in the model group. According to the multivariate Cox regression analyses, there were 13 lncRNAs identified as independent biomarkers for disease free survival in GC patients. The relevant estimated regression coefficients of these 13 prognostic lncRNAs were showed in Table 2. Therefore, a prognostic signature was conducted according to the following formula: Prognostic signature score = (− 0.712*GAS5-AS1) + (− 0.725*AL109615.3) + (− 0.830*KDM7A-DT) + (− 0.837*AP000866.2) + (− 0.984*KCNJ2-AS1) + (− 1.150*LINC00656) + (0.764*LINC01777) + (0.769*AC046185.3) + (0.775*TTTY14) + (0.783*LINC01526) + (0.803*LINC02523) + (0.941*LINC00592) + (1.162*C5orf66). According to the multivariate Cox regression analyses, a prognostic nomogram for prediction of disease free survival in gastric cancer patients was presented in Fig. 2.

**Table 2:** The model information of prognostic lncRNA predictors in univariate and multivariable Cox regression analyses

Variables	Univariate analysis			Multivariate analysis			
	HR	95% CI	*P*-value	Coefficient	HR	95% CI	*P*-value
GAS5-AS1 (high/low)	0.580	0.387–0.870	0.008	− 0.712	0.491	0.319–0.755	0.001
AL109615.3 (high/low)	0.632	0.422–0.948	0.027	− 0.725	0.485	0.315–0.744	0.001
KDM7A-DT (high/low)	0.628	0.420–0.939	0.024	− 0.830	0.436	0.275–0.690	< 0.001
AP000866.2 (high/low)	0.633	0.423–0.947	0.026	− 0.837	0.433	0.276–0.677	< 0.001
KCNJ2-AS1 (high/low)	0.642	0.429–0.959	0.031	− 0.984	0.374	0.237–0.588	< 0.001
LINC00656 (high/low)	0.651	0.436–0.973	0.036	− 1.150	0.317	0.199–0.503	< 0.001
LINC01777 (high/low)	1.907	1.260–2.886	0.002	0.764	2.147	1.354–3.403	0.001
AC046185.3 (high/low)	1.566	1.049–2.339	0.028	0.769	2.157	1.373–3.388	0.001
TTTY14 (high/low)	1.786	1.191–2.678	0.005	0.775	2.170	1.412–3.333	< 0.001
LINC01526 (high/low)	1.669	1.115–2.500	0.013	0.783	2.188	1.397–3.425	0.001
LINC02523 (high/low)	1.890	1.252–2.852	0.002	0.803	2.232	1.373–3.625	0.001
LINC00592 (high/low)	1.594	1.066–2.382	0.023	0.941	2.563	1.658–3.963	< 0.001
C5orf66 (high/low)	1.505	1.005–2.254	0.047	1.162	3.195	2.001–5.105	< 0.001

The medians of lncRNA expression values were used as cut-off values to stratify lncRNA expression values into high expression group (as value 1) and low expression group (as value 0)

HR, hazard ratio; CI, confidence interval

This nomogram could be used to predict the individual mortality risk. The value of each lncRNA was calculated according to the corresponding scale. The total score was obtained by adding the values of these thirteen lncRNA. The total score was projected to the probability of DFS in 1 year, 3 year, and 5 year respectively.

### Distribution characteristics of prognostic signature score

**Fig. 3:**
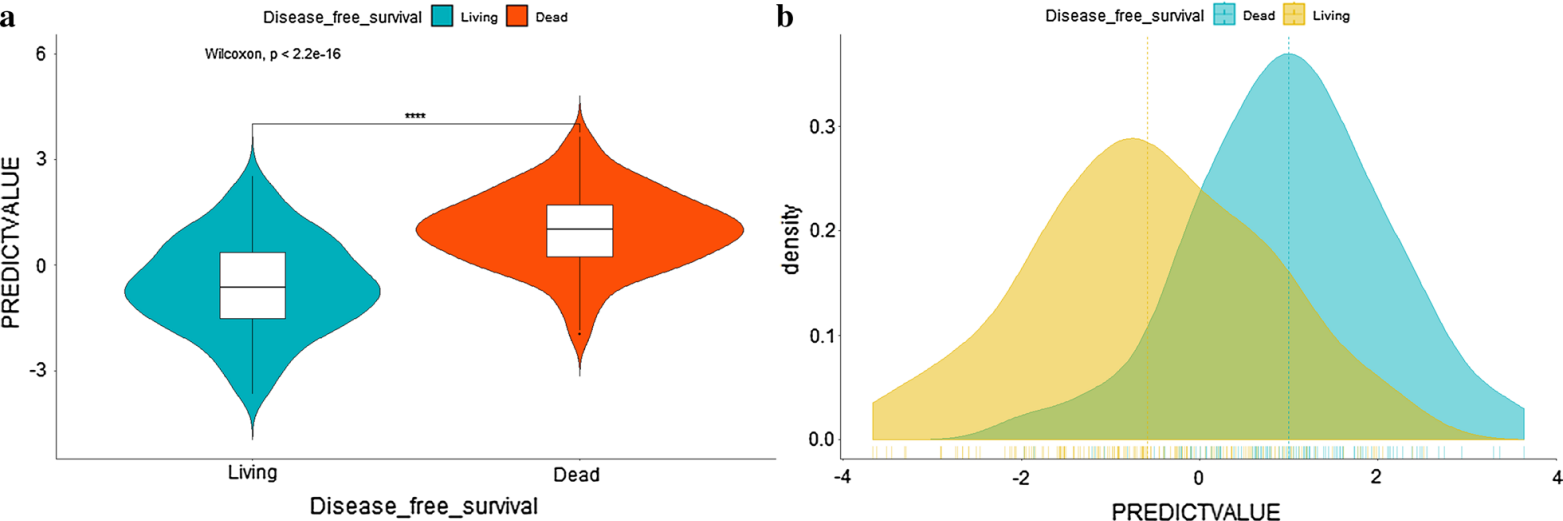
Distribution characteristics of prognostic signature score in the model group: the violin plot of prognostic signature score (**a**); the density plot of prognostic signature score (**b**)

**Fig. 4:**
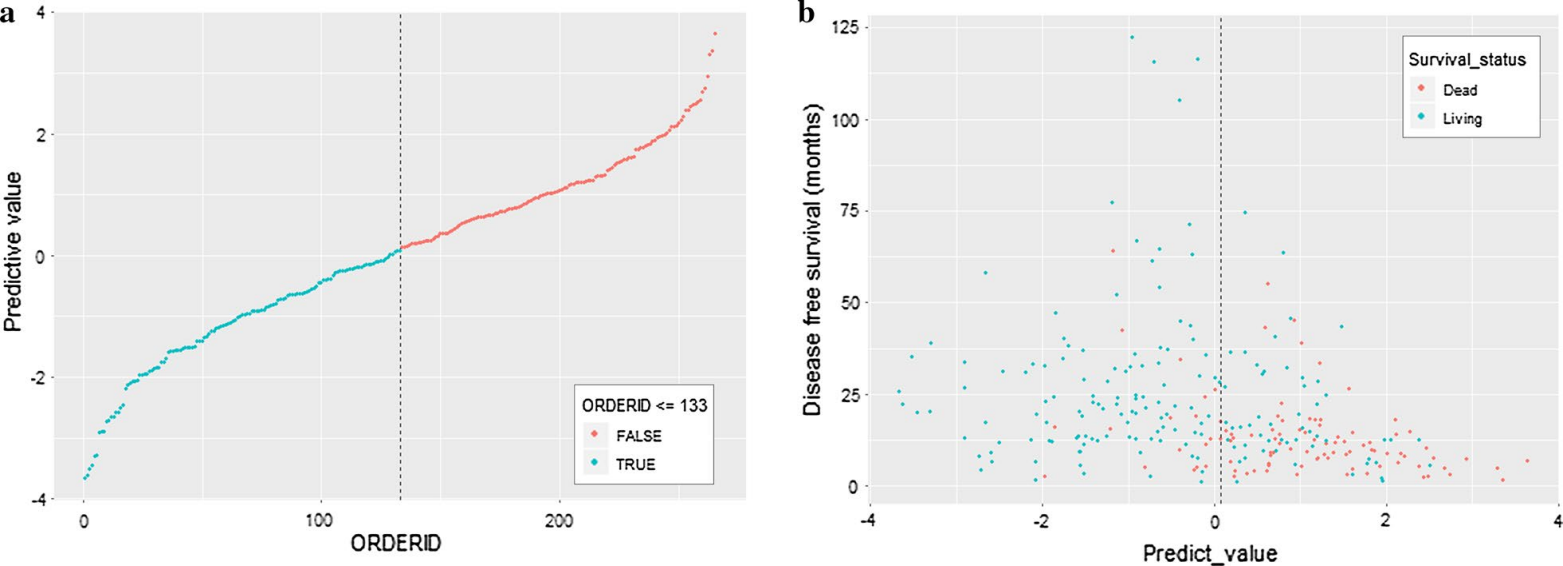
The distribution of prognostic signature score in the model group (**a**); the disease free survival status and disease free survival time in the model group (**b**)

For displaying the distribution characteristics of prognostic signature score, the violin plot (Fig. 3a), density plot (Fig. 3b), scatter plot (Fig. 4a), the interaction distribution scatter plot among DFS time, DFS status, and predictive value (Fig. 4b) were presented in Figs. 3 and 4.

### Clinical utility of prognostic signature

**Fig. 5:**
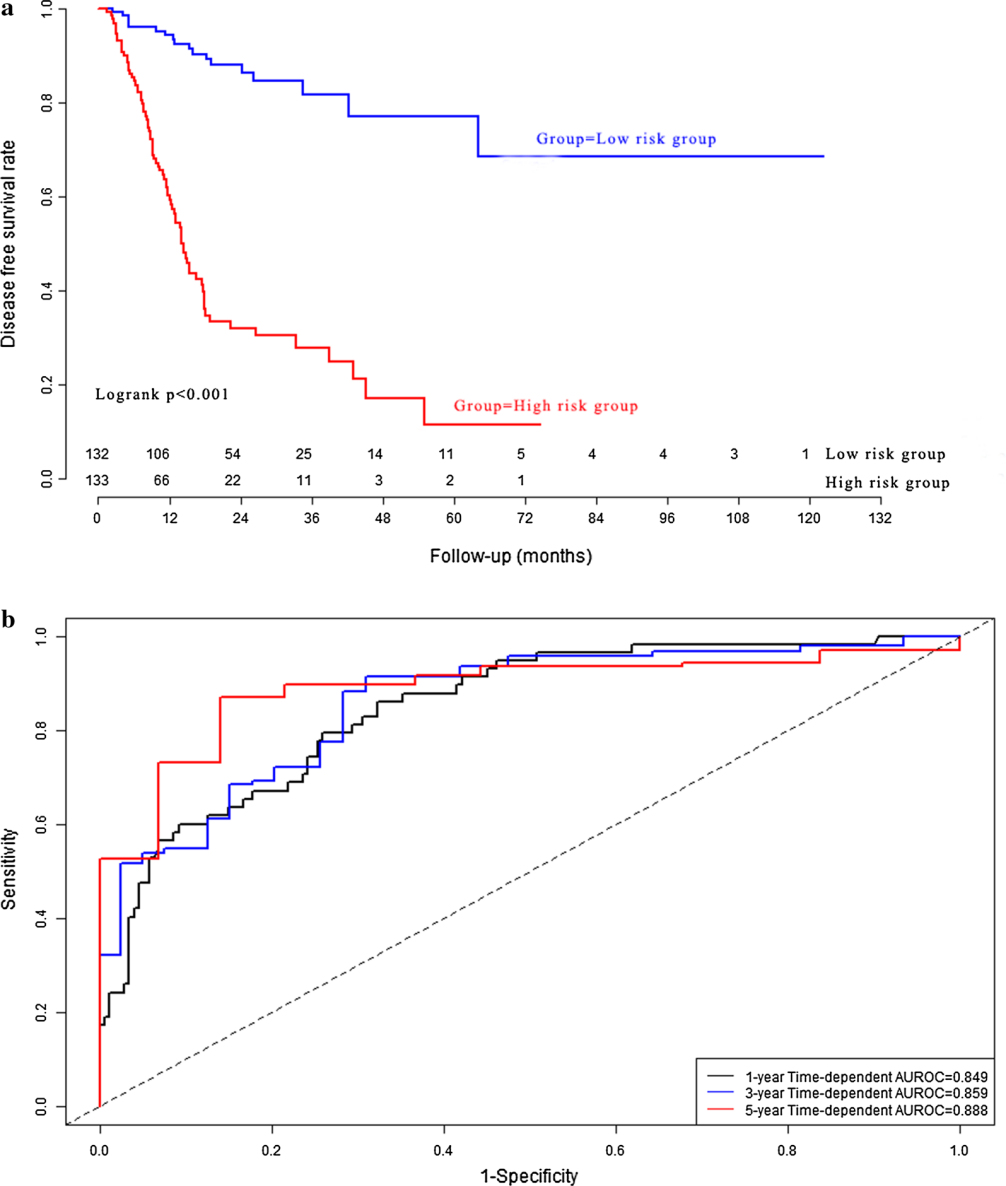
Performance of prognostic signature in the model group: the survival curves of gastric cancer patients (**a**); time-dependent receiver operating characteristic curves (**b**)

**Fig. 6:**
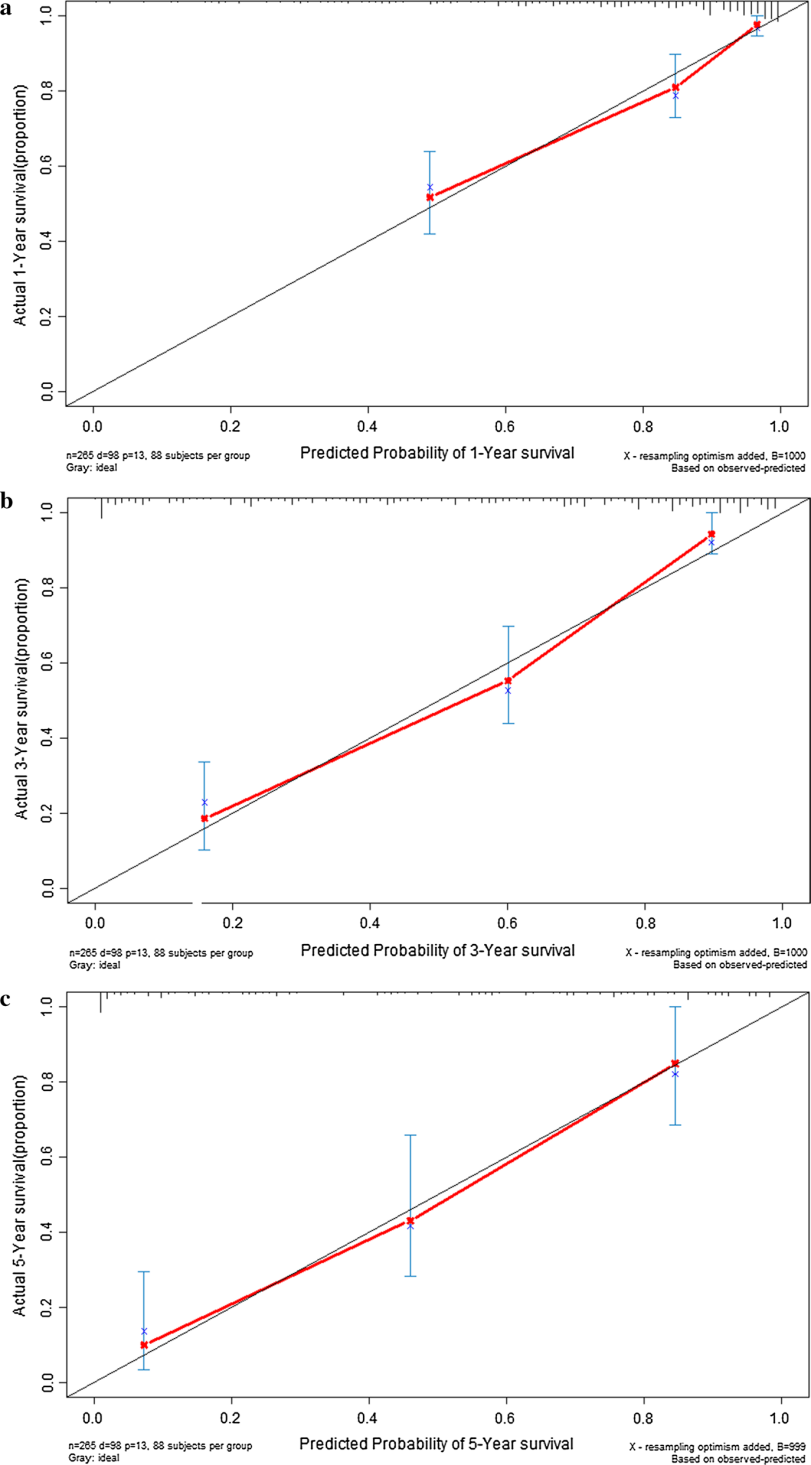
Calibration curve of prognostic signature in the model group: calibration curve for 1-year disease free survival (**a**); calibration curve for 3-year disease free survival (**b**); calibration curve for 5-year disease free survival (**c**)

According to the median value of prognostic signature score, GC patients in model cohort (n = 265) were stratified into high risk group (n = 133) and low risk group (n = 132). The disease free survival rate (Fig. 5a) in high risk group was significantly poorer than that in low risk group (*P *< 0.001). The cumulative proportion surviving at 1-year, 3-year, and 5-year were 94.3%, 81.7%, and 77.1% in low risk group, whereas it were 59.2%, 27.8% and 11.3% in high risk group respectively (all *P *< 0.001). The Harrell’s concordance indexes (C-indexes) of prognostic signature for disease free survival in the model group were 0.849 (95% CI 0.803–0.895), 0.859 (95% CI 0.813–0.905) and 0.888 (95% CI 0.842–0.934) for 1-year disease free survival, 3-year disease free survival and 5-year disease free survival respectively (Fig. 5b). The calibration curves for 1-year, 3-year, and 5-year disease free survival were presented in Fig. 6.

### External validation of prognostic signature

**Fig. 7:**
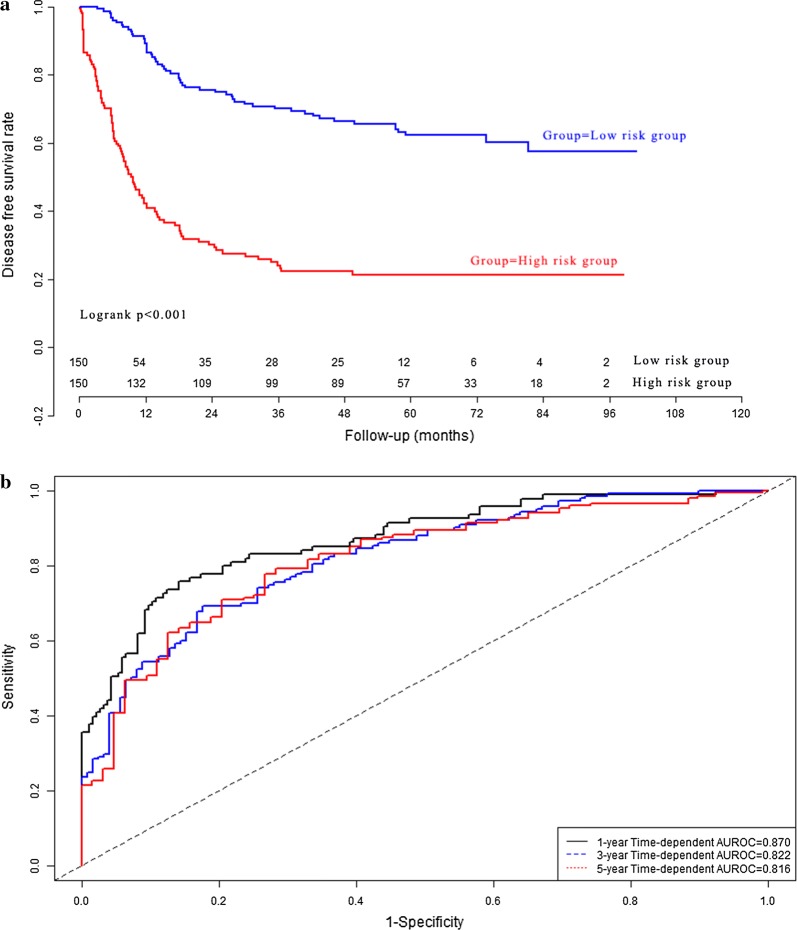
Performance of prognostic signature in the validation group: the survival curves of gastric cancer patients (**a**); time-dependent receiver operating characteristic curves (**b**)

**Fig. 8:**
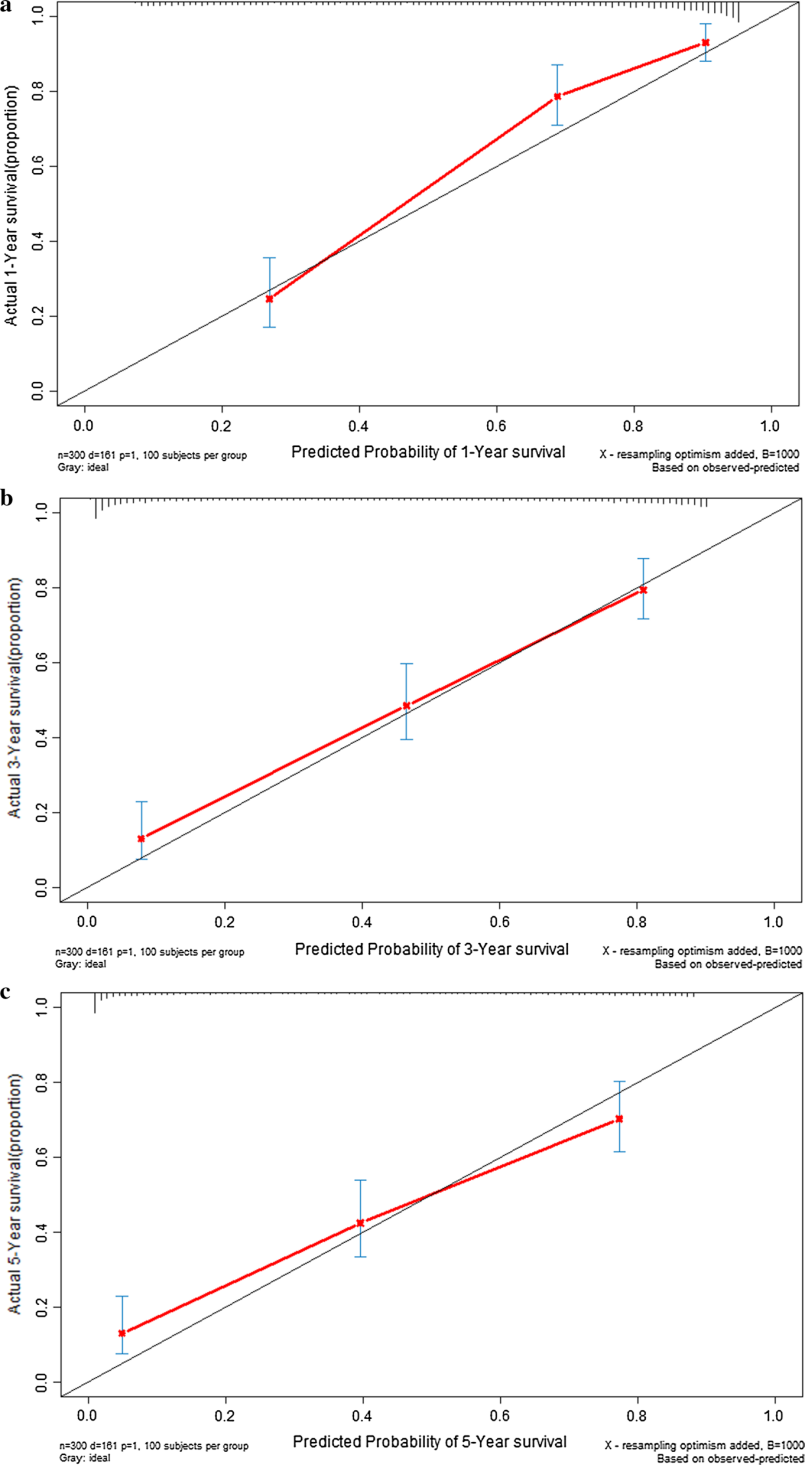
Calibration curve of prognostic signature in the validation group: calibration curve for 1-year disease free survival (**a**); calibration curve for 3-year disease free survival (**b**); calibration curve for 5-year disease free survival (**c**)

We validated the clinical utility of prognostic signature by using GSE62254 dataset. The prognostic signature scores for GC patients in validation dataset were calculated according to the previous prognosis signature formula in model dataset. Then the GC patients in validation set (n = 300) were divided into low risk group (n = 150) and high risk group (n = 150). The cumulative proportion surviving at 1-year, 3-year, and 5-year were 89.3%, 70.1%, and 62.3% in low risk group, whereas it were 42.4%, 24.1% and 21.4% in high risk group respectively (all *P *< 0.001). Kaplan–Meier analysis (Fig. 7a) indicated that there was significant difference in term of DFS between low risk group and high risk group in validation set (*P *< 0.001). The C-index of prognostic signature for disease free survival in the validation group were 0.870 (95% CI 0.834–0.906), 0.822 (95% CI 0.786–0.858) and 0.816 (95% CI 0.780–0.894) for 1-year, 3-year, and 5-year disease free survival respectively (Fig. 7b). The calibration curves for 1-year, 3-year, and 5-year disease free survival were presented in Fig. 8 for validation cohort.

### Independence assessment for prognostic significance of prognostic signature

We carried out multivariate Cox regression analyses to explore whether prognostic signature was independent to other clinical parameters for DFS in GC patients. The pathological diagnosis was performed according to the suggestions of American Joint Committee on Cancer (AJCC). Table 3 indicated that prognostic signature was an independent risk factor for DFS after adjustment for confounding effects of gender, age, and pathological stage in the model group. In the validation group, multivariate Cox regression analyses demonstrated that prognostic signature, age, and AJCC stage were independent risk factors for DFS.

**Table 3:** Univariate and multivariable Cox regression analyses for independence assessment of prognostic signature

	n	Univariate analysis			Multivariate analysis			
		HR	95% CI	*P*-value	Coefficient	HR	95% CI	*P*-value
*Model group (n = 265)*								
Age (high/low)	265	0.946	0.636–1.408	0.784	0.522	1.686	1.103–2.577	0.016
Gender (male/female)	265	1.872	1.173–2.988	0.009	0.463	1.589	0.992–2.547	0.054
AJCC PT (T4, T3/T2, T1)	265	1.385	0.876–2.191	0.164	− 0.084	0.919	0.546–1.547	0.752
AJCC PN (yes/N0)	265	1.639	1.034–2.599	0.036	0.121	1.128	0.596–2.135	0.711
AJCC PM (yes/M0)	265	1.056	0.512–2.178	0.883	0.208	1.231	0.574–2.637	0.594
AJCC stage (IV, III/II, I)	265	1.607	1.072–2.409	0.022	0.234	1.264	0.690–2.314	0.448
Prognostic signature (high/low)	265	7.355	4.378–12.356	< 0.001	2.098	8.148	4.718–14.071	< 0.001
*Validation group (n = 300)*								
Age (high/low)	300	1.356	0.995–1.849	0.054	0.400	1.491	1.090–2.041	0.012
Gender (male/female)	300	0.997	0.719–1.383	0.986	0.134	1.144	0.818–1.599	0.432
AJCC PT (T4, T3/T2, T1)	300	2.200	1.613–3.000	< 0.001	0.122	1.130	0.776–1.647	0.523
AJCC PN (N2, N1/N0)	300	3.023	1.542–5.927	< 0.001	0.378	1.459	0.712–2.987	0.302
AJCC PM (MX, M1/M0)	300	3.553	2.305–5.478	< 0.001	0.410	1.507	0.950–2.392	0.081
AJCC stage (IV, III/II, I)	300	3.410	2.366–4.915	< 0.001	0.977	2.655	1.652–4.267	0.000
Prognostic signature (high/low)	300	3.919	2.817–5.453	< 0.001	1.236	3.443	2.435–4.866	< 0.001

The median of prognostic signature scores was used as the cut-off value to stratify gastric cancer patients into high risk group and low risk group

AJCC, the American Joint Committee on Cancer; HR, hazard ratio; CI, confidence interval

### Functional enrichment analyses

**Fig. 9:**
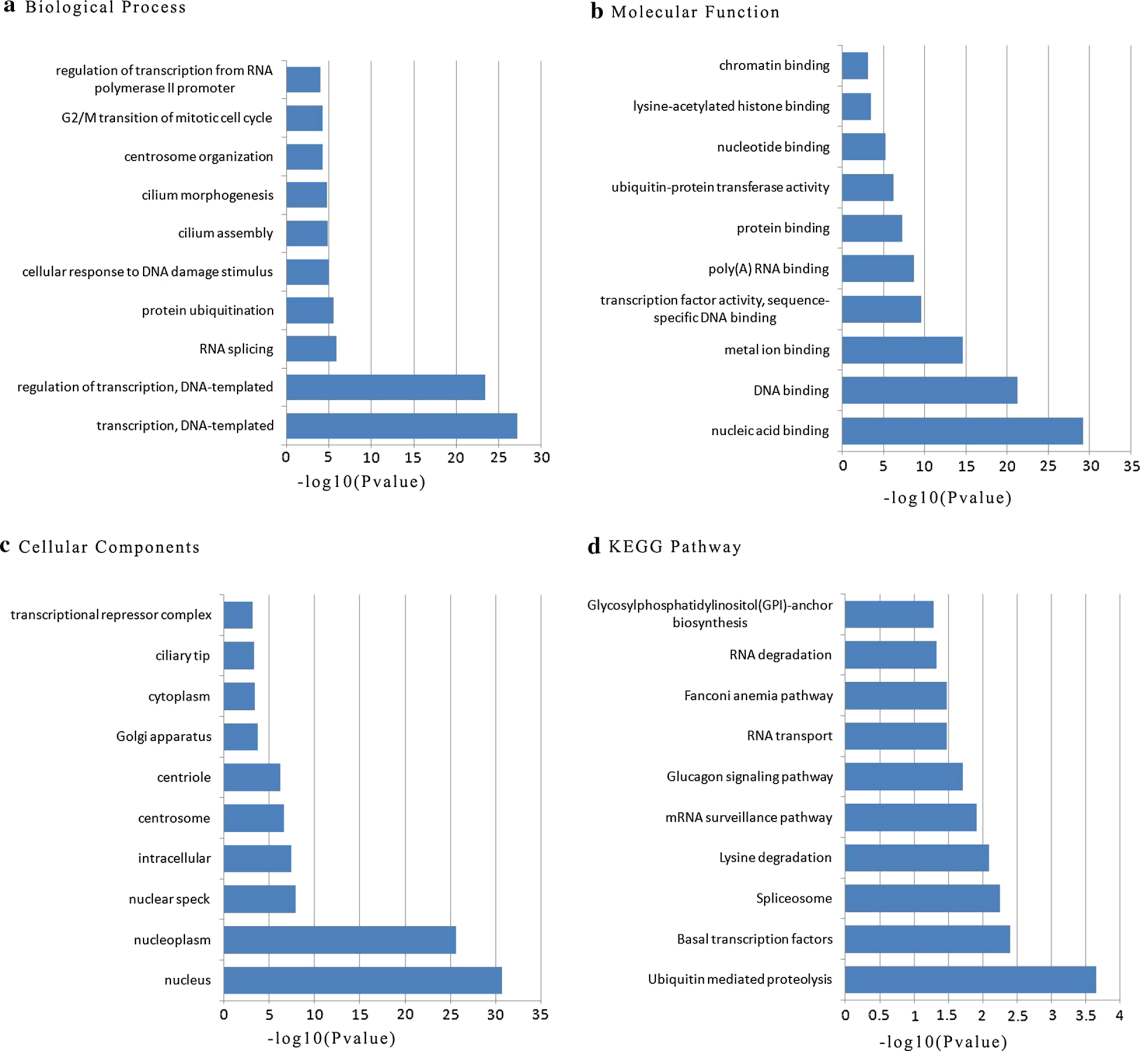
Functional enrichment analysis of prognostic signature: (**a**) biological process; (**b**) molecular function; (**c**) cellular component; (**d**) KEGG pathway. KEGG, Kyoto Encyclopedia of Genes and Genomes

According to a threshold of *P* value < 0.05 and |spearman correlation coefficient| > 0.5, there were 1280 mRNA genes that significantly co-expressed with the prognostic lncRNAs in the prognostic signature. Functional enrichment analyses were carried out through the Database for Annotation, Visualization, and Integrated Discovery (DAVID) Bioinformatics Resources (https://david.ncifcrf.gov/). The gene ontology (GO) biological process enrichment analyses and the Kyoto Encyclopedia of Genes and Genomes (KEGG) signaling pathways were presented in Fig. 9. Functional enrichment analyses of the 1280 co-expressed mRNA genes demonstrated that the co-expressed mRNA genes were mainly enriched in regulation of transcription, RNA splicing, protein ubiquitination, cellular response to DNA damage stimulus, cilium assembly, cilium morphogenesis, centrosome organization, G2/M transition of mitotic cell cycle, regulation of transcription from RNA polymerase II promoter.

### The decision curve analysis (DCA)

**Fig. 10:**
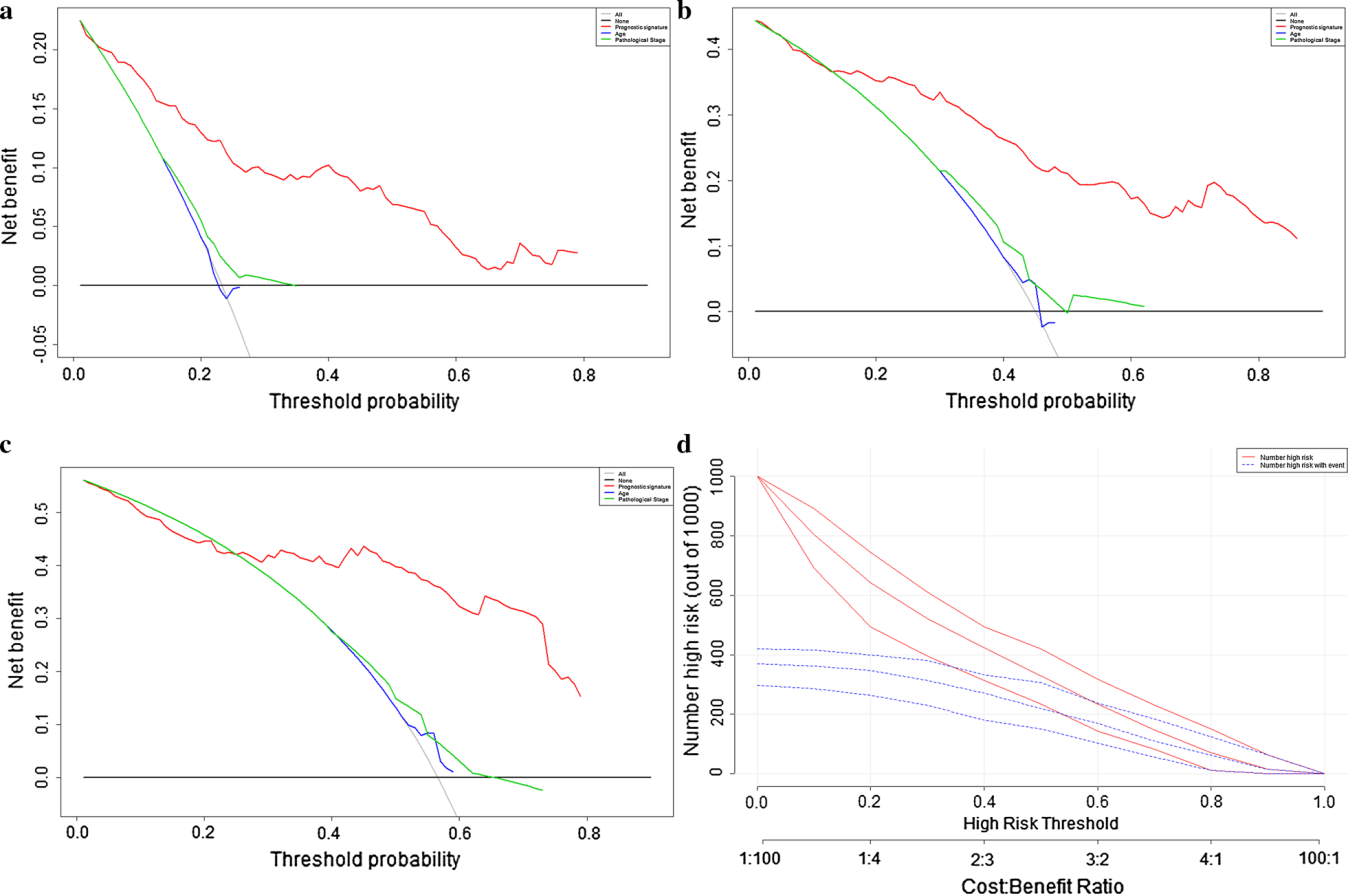
The decision curve analysis of prognostic signature: decision curve analysis for 1-year disease free survival (**a**); decision curve analysis for 3-year disease free survival (**b**); decision curve analysis for 5-year disease free survival (**c**). The y-axis represented the net benefit. The red solid line represented the prognostic signature. The grey solid line represented the net benefit of treating all patients within 1-, 3-, and 5-year. The black solid line represented the net benefit of treating no patients within 1-, 3-, and 5-year

As shown in Fig. 10, the prognostic signature (red line) had the higher net benefit than the pathological stage (green line). The decision curve analyses indicated that prognostic signature could gain more benefit than either the dead-all-patients scheme or the dead-none-patients scheme for prediction of 1-year DFS (Fig. 10a), 3-year DFS (Fig. 10b), and 5-year DFS (Fig. 10c). Clinical impact curve (Fig. 10d) depicted the prediction of risk stratification of 1000 patients by using resample bootstrap method. “Number high risk” indicated the number of patients classified as positive (high risk) by prognostic signature according to various threshold probabilities. “Number high risk with event” was the true positive patient number according to various threshold probabilities.

### Ten-group risk stratification chart

**Fig. 11:**
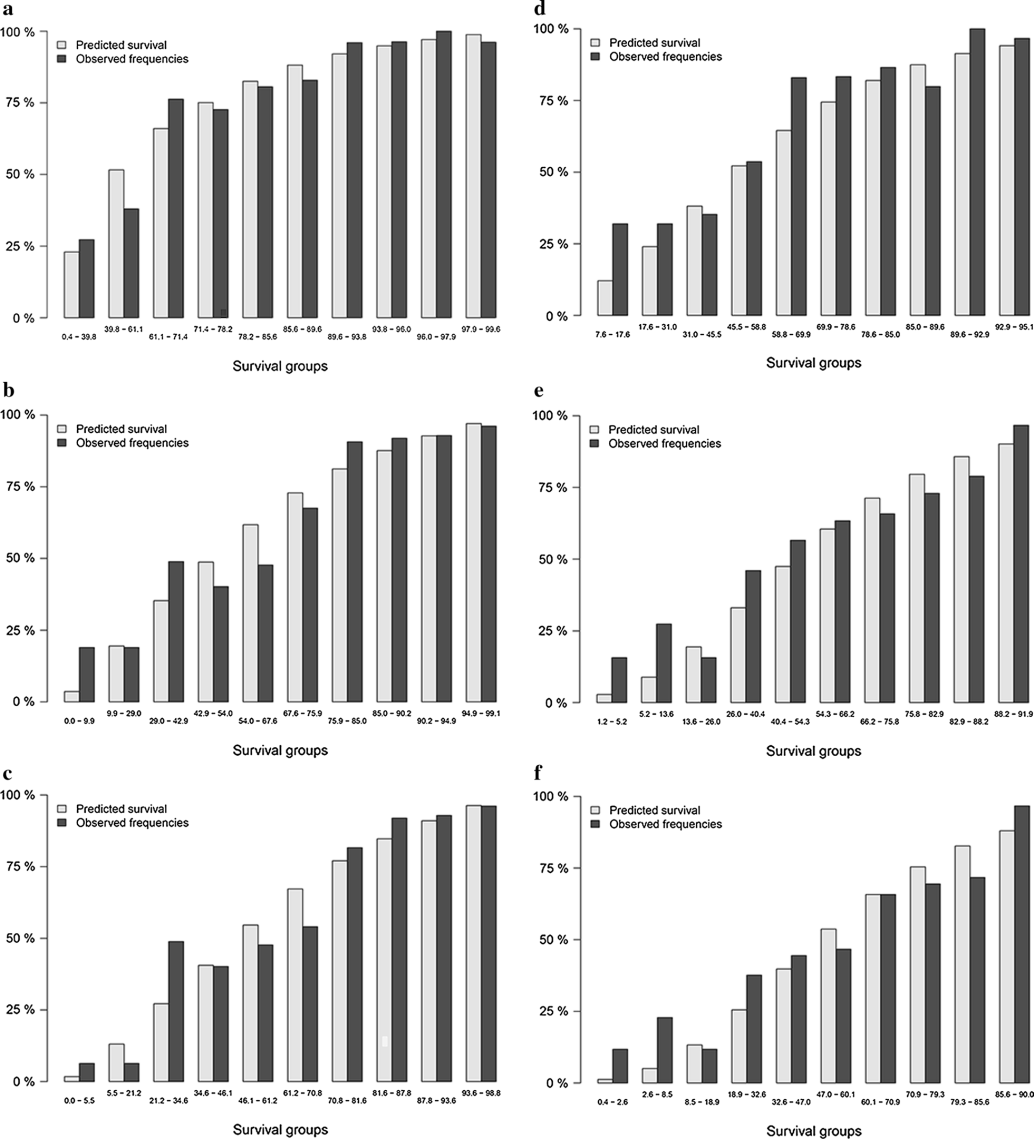
Ten-group risk stratification chart: (**a**) for 1-year disease free survival in model group; (**b**) for 2-year disease free survival in model group; (**c**) for 3-year disease free survival in model group; (**d**) for 1-year disease free survival in validation group; (**e**) for 2-year disease free survival in validation group; (**f**) for 3-year disease free survival in validation group

To explore the predictive performance of prognostic signature for DFS, a 10-group risk stratification chart was presented in Fig. 11. For model cohort, the discriminative ability of prognostic signature for 1 year, 2 year, and 3 year DFS were showed in Fig. 11a–c. For validation cohort, the discriminative ability of prognostic signature for 1 year, 2 year, and 3 year DFS were showed in Fig. 11d–f. Figure 11 demonstrated that patients in lower risk groups had higher survival probability and patients in higher risk groups had lower survival probability.

## Discussion

Accurate and reliable prognostic prediction is of critical importance for individualized treatment decision-making of GC patients. The current study developed and validated a thirteen-lncRNA prognosis signature for prognostic prediction of GC patients. The thirteen-lncRNA prognostic signature was proved to be helpful for individual mortality risk prediction and risk stratification of GC patients in an independent external dataset.

In the current study, the thirteen-lncRNA prognosis signature scores were calculated for prediction of DFS in GC patients in both model set and validation set. Poorer DFS were significantly related with high prognosis signature scores in both model set and validation set, demonstrating that the clinical performance of prognosis signature was stable and reliable for prognostic prediction of GC patients. Multivariate Cox regression analyses demonstrated that prognostic signature was an independent risk factor for DFS in both model set and validation set. Thus the thirteen-lncRNA prognosis signature was helpful to identify the patients with high mortality risk and improve the individualized clinical decision-making of GC patients.

The current prognostic signature has a good prospect for clinical application. All parameters in the current prognostic signature were generated by gene detection method, indicating that this prognostic model is a noninvasive method and can be used before surgery. Through this prognostic model consisting of thirteen prognostic lncRNAs, doctors and patients can pre-estimate the risk of death in the next 5 years. This prognostic information is valuable for patients to decide whether to receive surgical treatment or not. Ten-group risk stratification chart in the current study demonstrated that patients in lower risk groups had higher survival probability and patients in higher risk groups had lower survival probability.

GAS5-AS1 was an independent prognostic factor for Hepatocellular carcinoma patients and could be considered as a potential prognostic biomarker [23]. The reduced GAS5-AS1 was significantly correlated with larger tumor, higher TNM stage, and lymph node metastasis for non-small cell lung cancer patients [24]. TTTY14 was significantly correlated with overall survival for GC patients and the prognostic value of TTTY14 was independent to other clinical features [25]. LINC00592 was a potential cancer related lncRNA in cervical cancer and might activate the cancer progression through the regulation of transcription or structural integrity [26]. LincRNA C5orf66-AS1 hypomethylation was significantly associated with overall survival in patients with the squamous cell cancer in the head and neck region [27]. The aberrant hypermethylation-mediated downregulation of C5orf66-AS1 might play an important role in gastric cardia adenocarcinoma tumorigenesis and might serve as a potential prognostic biomarker in predicting gastric cardia adenocarcinoma patients’ survival [28].

The previous three prognostic signatures calculated the risk scores by using original gene expression values generated on different gene detection platforms and different standardized methods. The different gene detection platforms and standardized methods reduced the repeatability of research results and hindered the clinical application of prognostic signatures in other population. Therefore the current study couldn’t carry out the previous three prognostic signatures due to the different gene detection platforms and standardized methods. To improve the clinical application of the current prognostic signature in other study population, the thirteen-lncRNA prognostic signature scores in the current study were calculated based on the converted dichotomous values. This dichotomous conversion was helpful to eliminate the obstacles of different detection platforms and standardization methods. Therefore, the thirteen-lncRNA prognostic signature was more suitable for clinical prognostic prediction than the previous three prognostic signatures.

### Advantages of the current study

Firstly, this prognostic nomogram can directly provide the individual mortality percentage forGC patients, which is important for improvement of individualized treatment decision-making. Secondly, the thirteen-lncRNA prognostic signature is easy to calculate and understand by users without medical knowledge and professional calculation tool. Thirdly, for patients without pathological diagnosis or unwilling to undergo surgery, the thirteen-lncRNA prognostic signature provides a simple non-invasive preoperative predictive method for prognosis of GC patients, which is of clinical significance for individualized clinical decision-making before surgery.

### Limitations of the current study

As a clinical study by using study datasets downloaded from public databases, the model dataset and validation dataset did not contain detailed study information of drug regimen and other postoperative treatments, which might influence the therapeutic effect and prognosis. Additionally, the results in the current study depended on gene mining approach and lacked evidences from clinical experimental researches. Therefore, it is necessary to carry out large prospective studies to elucidate the relationship between the prognostic lncRNA biomarkers and DFS in GC patients.

## Conclusion

Taken together, a simple noninvasive prognostic signature was established for preoperative prediction of disease free survival in GC patients. This prognostic signature might predict the individual mortality risk of disease free survival without pathological information and facilitate individualized treatment decision-making.

## Additional files

**Additional file 1.** Model cohort dataset.

**Additional file 2.** Validation cohort dataset.

## Data Availability

The datasets analyzed in the current study are provided as the additional documents in the end of the current article.

## Abbreviations

GC: gastric cancer
TCGA: The Cancer Genome Atlas
ROC: receiver operating characteristic
DFS: disease-free survival
lncRNA: long non-coding RNA
HR: hazard ratio
CI: confidence interval
AJCC: the American Joint Committee on Cancer
GEO: the Gene Expression Omnibus
SD: standard deviation
DCA: decision curve analysis
